# Give It to Me Straight Doc: A Medical Student Perspective on Effective Goal-Oriented Clinical Education

**DOI:** 10.7759/cureus.69343

**Published:** 2024-09-13

**Authors:** Adam S Mangold, Jacob P Barr, John McGeehan

**Affiliations:** 1 Department of Medicine, Cooper Medical School of Rowan University, Camden, USA

**Keywords:** assessment, clinical learning environment, clinical medical education, feedback, goal setting

## Abstract

The clinical years of medical school are a time when students navigate a new learning environment. Due to inexperience, discordance may exist between veteran attendings and students who do not have their bearings in this new setting. We propose a solution to strengthen the clinician-student relationship by promoting a culture of goal-oriented clinical education via a two-pronged approach. First, standardized learning objectives should be established for each clinical rotation. Second, and arguably more importantly, students should create individualized goals to complement these objectives and further their personal educational interests. Once a culture of goal-oriented clinical education is implemented, we believe students will navigate their clinical years with increased confidence and competence. In this piece, we discuss our personal attitudes toward why clinicians and students should set goals, how they can set them, and what these goals should include. Once goals are established, feedback must be provided to students to continue the learning process. This comes in the form of comments from supervising preceptors who focus on areas of interest identified by the student, as well as from the results of standardized assessments. We passionately believe that together, the synergism of goal setting, feedback, and assessment creates a perfect mixture conducive to the formation of a positive learning environment.

## Editorial

The beginning of clinical training for medical students is filled with curiosity and excitement. At the same time, newcomer students enter a house of seasoned professionals, some more seasoned than others, in a heterogeneous mixture at the resident, fellow, and attending levels. Student inexperience results in a struggle to adjust to the new learning environment, and we believe this leads to diminished returns in learning.

One potential solution to strengthen the clinician-student relationship is to establish and promote a culture of goal-oriented clinical education via a two-pronged approach. First, a set of standardized learning objectives should be developed for each clinical rotation to ensure students have varied opportunities for patient care involvement. These objectives should be provided to students prior to each rotation and should be enforced by course directors throughout the clerkship and academic year. Second, and arguably more importantly, students should create individualized goals to complement these standardized objectives and further their personal interests. We hope that students will take charge of their own learning and pursue these individual goals with the help of their attendings and residents. By crafting and fostering a culture of goal-oriented clinical education, students can navigate the third and fourth years of medical school with increased confidence, competence, and ownership.

To properly frame our insights in context, we must recognize our inherent biases in crafting this work. After three years of collegiality and friendship, we began writing our perspectives to encourage dialogue on optimal medical educational strategies. We believe in the educator-student partnership's power to facilitate high-quality education. To this end, we have met an abundance of incredible educators at our home institution in Camden, New Jersey who strongly advocate for this mutual relationship between teacher and learner. Our experiences with these faculty have informed our perspectives, and we could not be more thankful for their efforts to develop us into compassionate, humanistic physicians. Our experiences may differ from students at different institutions, as we learn in an underserved, urban environment. While the setting of medical education may differ from ours, we do not believe this invalidates the overarching themes outlined here. We encourage readers to consider our perspectives in the context of their own learning environment.

As such, it is also important to recognize that our own specialties of interest will surely influence the opinions expressed here, with Mr. Barr entering Emergency Medicine and Mr. Mangold entering Anesthesiology. Although we have focused interests, we have completed our institution’s slate of third-year core rotations and feel that we have an appropriate understanding of our curriculum to provide these perspectives.

The purpose of this article is not to criticize attendings and residents unjustly. Instead, we strive to provide medical educators with a student perspective regarding effective clinical instruction. A training program's objective should be to provide an optimal, positive learning environment for all trainees regardless of background and ability. We hope the perspectives shared in this article will allow institutions to move closer to accomplishing this goal.

Why set goals, how should we set them, and what do they include?

Why Set Goals?

Goal setting in medical education allows students and faculty preceptors to focus on clinical experiences that reinforce learning points. Research shows that medical students value goal setting, with goal-oriented feedback being particularly important for growing and learning. Formative feedback during rotations allows students to recognize strengths and weaknesses to improve on throughout the remainder of the course [1]. They can realign their personal goals toward improving these weaknesses, and work on becoming more receptive to preceptor feedback [2]. It is also important for students to feel comfortable providing feedback to attendings and residents. When students are trained in giving specific, constructive feedback to faculty, they can more effectively communicate learning goals and potential gaps in learning. This feedback allows preceptors to adjust their teaching styles to better meet student needs and improve the educational experience [3].

Setting educational goals is not simple and requires extensive communication between faculty and students. Farrell et al. followed five clinician-educators longitudinally who incorporated goal-setting and goal-oriented feedback sessions into their practice. They found that although improved faculty-learner rapport and appreciation for student efforts were present, there was still significant discord between preceptor and student goals. At times, these goals did not match, or students were seeking to accomplish goals not achievable within the constraints of the rotation [4]. It is important to reconcile attending and student goals to craft a cohesive educational plan for the limited time in each learning environment. Stoddard et al. recently discussed a new, revolutionary approach to medical school curriculum development termed “co-creation” [5]. Curricular “co-creation” is described as a “bi-lateral partnership” between faculty and students, where students now have a larger say in creating goals for their own medical education [5]. The authors astutely report that “co-creation” can improve learning and reform the medical profession by driving it away from its archaic, hierarchical roots [5]. We concur with this perspective and believe students should have the ability to tailor their own learning goals based on clinical interests. Students should engage in “co-creation” with faculty to ensure they have educational experiences that champion the accomplishment of their goals in a time-efficient, directed manner.

How Should We Set Them?

Creating achievable goals for medical students during their clinical years starts at the top of the administrative chain with course directors. We believe it is the duty of course directors to “set the stage” for rotations in their syllabi and orientation sessions. This entails describing rotation workflow, student responsibilities, and standardized learning goals.

It has been clear for years that well-crafted learning objectives are important when developing an effective clinical curriculum [6]. The concept of delivering specific, increasingly complex, and targeted learning objectives originated with Bloom’s taxonomy and continues to be refined today for those in medical education [7,8]. The literature provides many recommendations for the development of medical student clinical learning objectives. Some guides are institution-focused and do not make any specific references to “student goals” or “learner goals,” instead using a hypothetical “typical” medical student as a roadmap for generic curriculum design [9]. Other guides provide detailed, sometimes specialty-specific, instructions that describe how to write learning objectives, why writing them is important, and how to effectively implement them [10,11]. We believe these specific guides are better suited for creating an environment that promotes the accomplishment of student-developed clinical goals. Medical school leadership should also support course directors with didactic workshops focused on writing these objectives and syllabi that adequately expose students to their respective specialties. Faculty should not be expected to “set the stage” on their own without prior administrative support and training.

Once the “stage has been set,” attendings and residents in each department should emphasize the teaching points laid out by course directors. It is important to note that goals of learning should be different from specialty to specialty and should be framed around typically seen patient concerns for the respective rotation. Daily variation will exist within a given rotation as well; surgical rotations may emphasize specific pathologies or operations, while clinic days may have a wider scope of multiple disease processes.

What Do They Include?

We believe learning goals should involve clinical exposure that emphasizes prime teaching points with patient encounters. This can include focusing on common pathologies and patient concerns or practicing skills frequently used in procedural specialties (i.e., knot-tying or suturing). Students should own their patients and propose care decisions for them, with appropriate input from residents and attendings. We also advocate for student involvement in procedures as permissible, without compromising patient safety and high-quality healthcare. Additionally, without beratement, students should engage in academic lines of questioning with preceptors. Classically, this was referred to as “PIMPing” (“put in my place”) and had a negative connotation. If lines of questioning are pursued with the intent of emphasizing teaching points and engaging students in the learning process, we see no problem with this.

We also believe future physicians will encounter increasingly diverse patient populations and should receive extensive diversity education. The literature supports this belief, encouraging encounters with patients of various backgrounds, which will help providers develop the skills necessary for working with diverse populations [12]. Students should actively set goals in diversity education and seek out a wide variety of patient encounters. This variety in patient encounters has been shown to be key in medical student learning, providing a rewarding and challenging learning environment that promotes increased clinical competency and confidence [13].

Our vision is to have teaching attendings and residents engage with students in five-minute scrums that focus on desired learning objectives. If possible, these objectives will be incorporated into the team’s daily workflow. One option is for students to craft a card with weekly goals, which can then be provided to preceptors who will monitor for adequate learning opportunities addressing these goals. Perceived student strengths and weaknesses can then be longitudinally tracked and addressed during feedback sessions. Attendings should also provide direct, straightforward, achievable goals to students that are well-accomplishable within a given time. These may or may not reflect the goals set forward by course directors, as some attendings prefer to focus on certain patient concerns or diseases. Goals should be delivered in a direct manner to ensure student understanding without any necessary “mind reading” to determine what actions a learner should take. Students should always be encouraged to independently think through assessments and plans on their own. Since we are learners, it is important for attendings and residents to emphasize psychological safety, providing positive reinforcement for independent student thought and feedback for improvement.

There are many instruction methods presented in the literature that may reflect optimal ways for faculty to teach medical students. The manuscript authored by Burgess et al. most accurately supports and emphasizes our own beliefs on the value of clinical education. They remind us that the best clinical teachers are our own patients [14]. While simulation with standardized patients is an effective way to practice physical exam techniques and conversational skills before real clinical encounters, it cannot replace the benefits gleaned from the bedside [14]. The literature fully endorses their belief in three key learning domains that need to be integrated with bedside teaching to obtain maximum student benefit: clinical knowledge and skills, professionalism within the team and toward others, and communication abilities with staff and patients [14]. Students should aim to set achievable goals with faculty preceptors addressing these specific domains when providing care at the bedside. We also find other examples in the literature that extensively support student-directed learning and goal-setting focused on bedside teaching and the development of professionalism and communication skills [15-18].

Feedback and assessment

Since medical students constantly strive to perform well on rotations, it is important to ask: How can performance be tracked and evaluated? There must be methods to evaluate if students met their goals and how they worked toward them. Two main pedagogical tools used to evaluate whether medical students engaged well in the learning process are feedback from attendings and residents the student worked with, and formal written assessments, including shelf exams and the United States Medical Licensing Examination (USMLE) Step 2 CK (Clinical Knowledge) exam. Feedback and assessment are tied at the hip because of the essential need for both continued student and faculty improvement.

Clinician feedback is essential in knowledge and skill development for medical students [19-23]. While there is no comprehensive formula to provide feedback to students, we do believe certain guidelines should be followed. Feedback and criticism should always be constructive, and never demeaning or harming students. Feedback should assist students in achieving their predetermined goals and strengthening their skills, not impede progression in the curriculum. While it may be rotation-dependent, feedback could occur daily, weekly, and certainly at the halfway and end points of rotations. The preceptor providing feedback should make direct reference to the initial goals set by the student and address specific learner concerns. The feedback should also be more than a simple “student did well,” or “accomplished all tasks for the week.” We believe the ideal review of student performance is more of a qualitative description than a quantitative one. Instead of providing numerical scores, specific anecdotes and narratives should be shared with the student from preceptor observations. This is one of the most essential parts of the feedback process, as it provides students with positive reinforcement that they are performing well. It does not hurt that many of these narratives are also included in the Medical Student Performance Evaluation dean’s letters for residency programs to consider. There is extensive discussion of providing effective feedback in the literature, with one meta-analysis showing that providing feedback to students had a positive impact in over 75% of studies examined [24]. Many of the tips provided in the literature echo our sentiments, including providing regular, direct, constructive criticism that focuses on evaluating student-directed goals and ensuring learners have a plan moving forward for continued improvement [14].

For many students, the less exciting part of medical school centers on examination and assessment performance. Shelf exams taken after individual rotations evaluate knowledge obtained in each discipline. This experience culminates with the scored USMLE Step 2 CK exam at the end of the third/fourth year, which now has an arguably increased importance given the recent decision to make the USMLE Step 1 exam pass/fail. When given with the correct intention of evaluating students and providing feedback, assessments can determine student knowledge competencies, provide learners with content areas that require improvement, and determine if the content was delivered effectively. Our opinions are consistent with the literature, which also supports assessment as a tool to provide learners with feedback on strengths and weaknesses; so long as clear outcomes are defined, and the assessment is shown to be reliable, valid, and cost-effective [14,25-29].

Individual rotations will have their own methods for didactic delivery. We believe a balance between exam preparation and delivery of hands-on practical knowledge must be used to provide students with a quality education. Reviewing concepts that reflect high-yield shelf exams and USMLE Step 2 CK topics, including classic “buzzwords,” imaging findings, and diagnostic schemas will also serve students well clinically. An effective didactic curriculum will incorporate sufficient practice questions that allow students to apply concepts to higher orders of thinking. We also propose that preceptors ask students what topics they would like to cover in these sessions, allowing students to utilize study time wisely. Reviewing questions together will strengthen ideas of what potential pathologies could be and how they are worked up diagnostically.

Future directions

Overall, the goal of clinical education is to expose learners to the beautiful variety of ways to practice medicine. By doing so, students gain insight into their desires for practice when they grow up, and allow them to adequately prepare for residency. We believe attending physicians and residents have a responsibility to provide students with a high-quality education. They can do so by precepting students with objectives for learning, with feedback provided regularly. Our vision with this piece was to lay the groundwork for providing preceptors with the tools to understand why medical educators should set goals, how they should be set, and what these goals should encompass. The literature is currently sparse in terms of reporting the outcomes of effectively accomplishing student-generated goals in the clinical setting. We hope to inspire curriculum writers to encourage medical students to develop their clinical goals.

An important part of the learning process is obtaining feedback, as we believe this enables continuous improvement in student performance and faculty teaching abilities. Assessments, although loathed by students, provide another form of feedback regarding content mastery. Together, the synergism of goal setting, feedback, and assessment creates a perfect mixture conducive to the formation of a positive learning environment. All this advocacy from us begs the question, are we asking too much of our educators? Probably, and we acknowledge that upon completing residency and entering attendinghood, this piece may read poorly in hindsight as we end up *hoisted with our own petard* [30]. However, as medical students in the current learning environment, it is our duty to advocate for fellow students and ensure we maintain the highest standards of the American medical educational system through goal-oriented clinical experiences. Some medical schools have already implemented learner-directed goal setting in an open curriculum, although these students can struggle if they are unclear of grading criteria or expectations [31]. As a solution, peer-developed clinical guides have been used to properly introduce students to a given rotation, raising student confidence as the clinical year progressed [31]. This idea resonates strongly with us, as a guide written by fellow learners may be well-received by other students prior to starting new clerkships. To this effect, we will provide a future commentary featuring specific examples from our own experiences of how attendings and students can set learning goals in each clinical rotation.
